# Stabilisation of solid-state cubic ammonia confined in a glass substance at ambient temperature under atmospheric pressure†

**DOI:** 10.1039/d4ra00229f

**Published:** 2024-05-20

**Authors:** Masao Morishita, Hayate Miyoshi, Haruto Kawasaki, Hidefumi Yanagita

**Affiliations:** a National Institute for Materials Science (NIMS) (Formerly Department of Chemical Engineering and Materials Science), University of Hyogo Japan MORISHITA.Masao@nims.go.jp; b Department of Chemical Engineering and Materials Science, University of Hyogo Japan; c Sanalloy Industry Co., Ltd Japan

## Abstract

Ammonia, a widely available compound, exhibits structural transitions from solid to liquid to gas depending on temperature, pressure, and chemical interactions with adjacent atoms, offering valuable insights into planetary science. It serves as a significant hydrogen storage medium in environmental science, mitigating carbon dioxide emissions from fossil fuels. However, its gaseous form, NH_3_(g), poses health risks, potentially leading to fatalities. The sublimation pressure (*p*_sub_) of solid cubic ammonia, NH_3_(cr), below 195.5 K is minimal. In this study, we endeavoured to stabilise NH_3_(cr) at room temperature for the first time. Through confinement within a boric acid glass matrix, we successfully synthesised and stabilised cubic crystal NH_3_(cr) with a lattice constant of 0.5165 nm under atmospheric pressure. Thermodynamic simulations affirmed the stabilisation of NH_3_(cr), indicating its quasi-equilibrium state based on the estimated standard Gibbs energy of formation, 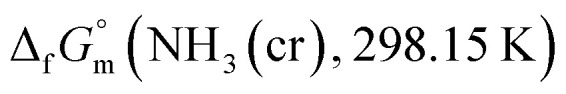. Despite these advancements, the extraction of H_2_(g) from NH_3_(cr) within the boric acid glass matrix remains unresolved. The quest for an external matrix with catalytic capabilities to decompose inner NH_3_(cr) into H_2_(g) and N_2_(g) presents a promising avenue for future research. Achieving stability of the low-temperature phase at ambient conditions could significantly propel exploration in this field.

## Abstract 2

Ammonia exhibits structural transitions depending on T and p, offering valuable insights into planetary science. Cubic crystal ammonia was stabilized at ambient T under atmospheric p for the first time, being a candidate of hydrogen storage medium.

## Graphical abstract

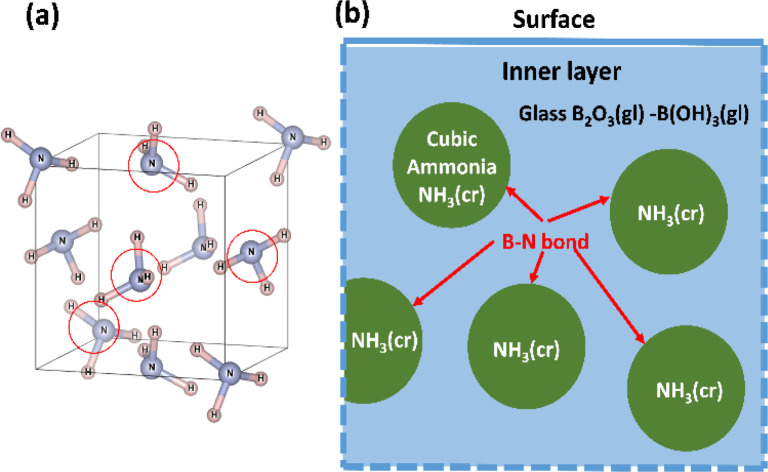

## Introduction

Ammonia, abundant throughout the cosmos, plays a crucial role in various celestial bodies. Within the depths of Uranus and Neptune, the presence of “hot ice” is notable, comprised of water, methane, and ammonia, sustained under high temperature and pressure. This compound also manifests as stoichiometric ammonia hydrates in many of the icy moons orbiting the outer planets.^2^ Moreover, in the atmospheric layers of Jupiter, ammonia functions as a significant chemical constituent.^3^ This cosmic ubiquity of ammonia underscores its relevance to the origins of life, as it constitutes an essential element of the planetary environments. Notably, in practical applications on Earth, ammonia serves as a foundational material for the production of urea fertilisers *via* the Haber–Bosch process, a critical measure in averting food crises.^4^ Furthermore, from an environmental science perspective, ammonia holds promise as a hydrogen storage medium for mitigating carbon dioxide emissions stemming from fossil fuel utilisation.^5–10^ The foundational understanding of ammonia spans disciplines such as space exploration, life sciences, material engineering, and environmental studies, primarily revolving around its stability across different phases: solid, liquid, and gas.^11–19^ Pressure–temperature diagrams detailing these phases have been meticulously constructed, underscoring the versatile nature of ammonia. For instance, its triple point, occurring at 195.5 K and 0.0609 bar, highlights the stability of its gas phase even at ambient temperatures under atmospheric pressure. Notably, our recent advancements have led to the synthesis of solid-state ammonia, adopting a cubic structure within boric acid through an innovative freeze-drying technique. This breakthrough opens avenues for further exploration at the forefronts of diverse scientific domains, hinting at promising implications and applications of cubic ammonia.^1–19^

Shifting perspectives towards utilising ammonia as a hydrogen storage material prompts consideration of the challenges associated with its storage and transport due to its harmful effects on human health. These effects encompass potential eye damage upon contact, respiratory distress upon inhalation, and the risk of impaired consciousness due to elevated blood ammonia levels, which can prove fatal in severe cases. Thus, this study delves into investigating small-scale and distributed mild-type ammonia production as a viable alternative.

To enhance the Harber–Boesch process,^4^ we explore the low-pressure synthesis of ammonia utilising lithium compound catalysts.^5^ Furthermore, alternative methods for ammonia production are investigated, including the cleavage of strong N–N bonds in N_2_(g) molecules through electrolysis,^6–8^ discharge processes,^9^ and surface plasmon resonance.^10^

Conversely, ammonia can be transformed into H_2_(g) and N_2_(g) with the aid of appropriate catalysts such as Ni,^20^ zeolites,^21^ and CaNH.^22^ The resulting H_2_(g) can then be separated using a membrane for efficient utilisation.^23^ Additionally, the solid-state cubic NH_3_(cr) with its low sublimation pressure presents another potential avenue for hydrogen storage and supply, particularly in small-scale and distributed mild-type production scenarios. Notably, this study marks the first description of the synthesis and thermodynamic analysis of cubic ammonia.

## Experimental

### Process design

The triple point of NH_3_ is characterised by specific temperature (195.5 K) and pressure (0.0609 bar) values.^11–19^ Below 195.5 K and at pressures exceeding 0.0609 bar, NH_3_ exists in its solid-state cubic form.^24,25^ Given this, achieving the stabilisation of solid-state cubic-NH_3_(cr) at ambient temperature and atmospheric pressure presents significant challenges. To address this obstacle, we developed a process design aimed at creating an embedded structure. This structure effectively confined NH_3_(cr) within a glass matrix, thereby enabling the stabilisation of NH_3_(cr) at ambient temperatures and under atmospheric pressure.

**Fig. 1:**
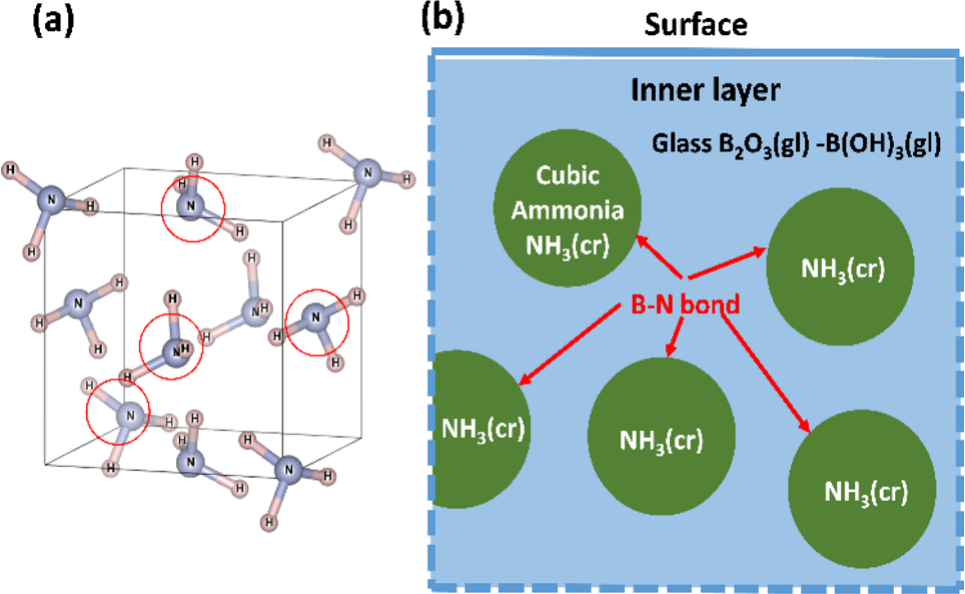
(a) Unit cell of cubic NH_3_(cr)^24–26^ composed of four molecules marked with open circle; (b) schematic model for stabilising the solid-state cubic ammonia (NH_3_(cr)) at ambient temperature, which is otherwise only stable below 195.5 K under atmospheric pressure.

Fig. 1(a) depicts a unit cell of cubic-NH_3_(cr),^24–26^ comprising four molecules denoted by open circles. Fig. 1(b) presents a schematic illustrating the process design. In this design, a boric glass shell serves as the matrix for confining the NH_3_(cr). The glass matrix, termed B_2_O_3_(gl)–B(OH)_3_(gl), is composed of boron trioxide (B_2_O_3_(gl)) and orthoboric acid (B(OH)_3_(gl)), synthesised by the dehydration of an aqueous solution resulting from the mixing of B_2_O_3_(cr) and pure water. Importantly, the geometric structure of NH_3_(cr) is confined within the B_2_O_3_(gl)–B(OH)_3_(gl) matrix.

It is noteworthy that in the cubic boron nitride (cBN) crystals, the covalent bond between a boron and a nitrogen atom is exceptionally strong. Similarly, a strong B–N bond^27^ is formed at the interface between the NH_3_(cr) and the B_2_O_3_(gl)–B(OH)_3_(gl) glass matrix. This robust interface is anticipated to stabilise the cubic structure of NH_3_(cr), ensuring stability under ambient temperature and atmospheric pressure conditions.

The freeze-drying technique was utilised to encapsulate cubic NH_3_(cr) within B_2_O_3_(gl)–B(OH)_3_(gl). Initially, a frozen mixture of ammonia and boric acid in water was prepared at 77 K through liquid-nitrogen cooling, wherein ammonia was trapped as frozen ammonium (NH_4_^+^ (cr)) ions within ice (H_2_O(cr)). Subsequently, NH_4_^+^(cr) needed to undergo a reaction with hydroxide ions (OH^−^(cr)) within the ice to achieve charge neutrality and enable sublimation into NH_3_(g). However, OH^−^(cr) mobility is restricted at low temperatures, resulting in condensation of NH_4_^+^(cr). Finally, the removal of the ice molecules (H_2_O(cr)) facilitated the containment of NH_3_(cr) within B_2_O_3_(gl)–B(OH)_3_(gl).

### Sample preparation

**Fig. 2:**
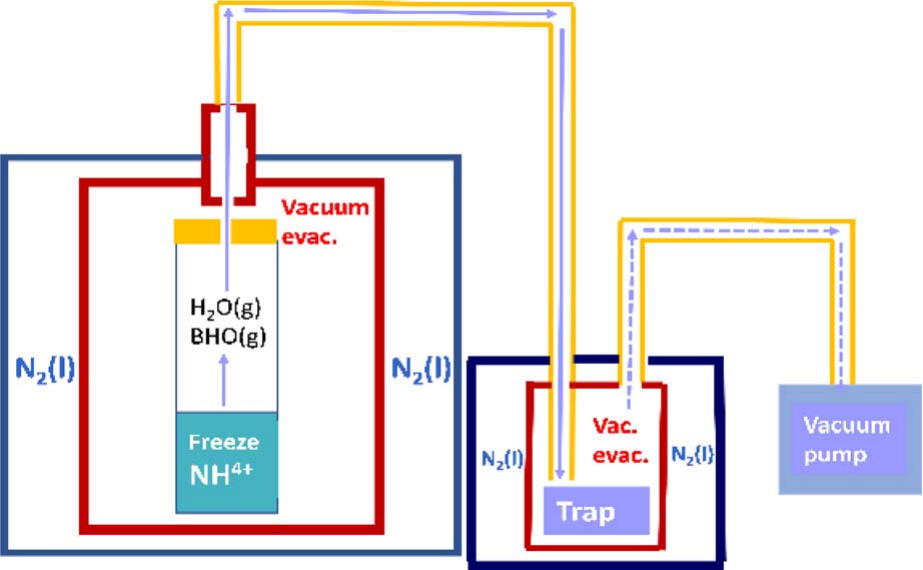
Schematic of freeze-drying apparatus and method for synthesising the solid-state cubic-NH_3_(cr).

Commercial aqueous ammonia solution (29 mass% NH_3_(aq.), Kanto Chemical Co., Ltd, Tokyo) and boron trioxide powder (B_2_O_3_(cr), 99.9%, Kojundo Chemical Laboratory Co., Ltd, Saitama) served as the starting materials. To ensure the stability of the NH_3_(cr) within the embedded structure, crucial for the formation of B–N bonds^27^ (Fig. 2), a 1 : 1 relative ratio of B to N was targeted. Thus, 160 mg of B_2_O_3_(cr) powder and 0.3 mL of 29 mass% NH_3_(aq.) were employed, equating to 4.6 × 10^−3^ moles for both B and N. Initially, the required amount of B_2_O_3_(cr) powder was placed in a test tube, followed by addition of NH_3_(aq.) to impregnate the B_2_O_3_(cr) powder. Subsequently, the test tube containing NH_3_(aq.)-impregnated-B_2_O_3_(cr) powder was sealed with a rubber cap, featuring a centrally punched hole (diameter 0.5 mm) for vacuum evacuation during the freeze-drying process.

Fig. 2 depicts a schematic of the apparatus utilised for freeze-drying. A test tube containing NH_3_(aq.)-impregnated B_2_O_3_(cr) powder, sealed with a rubber cap, was positioned within a custom-made copper reaction vessel. An exhaust port was integrated into the copper vessel to facilitate vacuum evacuation. Once the copper vessel, along with the test tube, was placed in a thermostatic bath, liquid nitrogen was employed to freeze the moisture, ammonium ions (NH_4_^+^(aq.)), and hydroxide ions (OH^−^(aq.)). Subsequently, after 1 h, frozen H_2_O(cr) was sublimated *via* vacuum evacuation for 2 h at 77 K, with NH_4_^+^(aq.) being condensed using a rotary vacuum pump. Following freeze-drying, the copper vessel was promptly removed to prevent moisture adsorption by the freeze-dried products (FD products) due to condensation. Evacuation using a vacuum pump persisted for 1 h at 295 K. Finally, the FD products were collected.

**Fig. 3:**
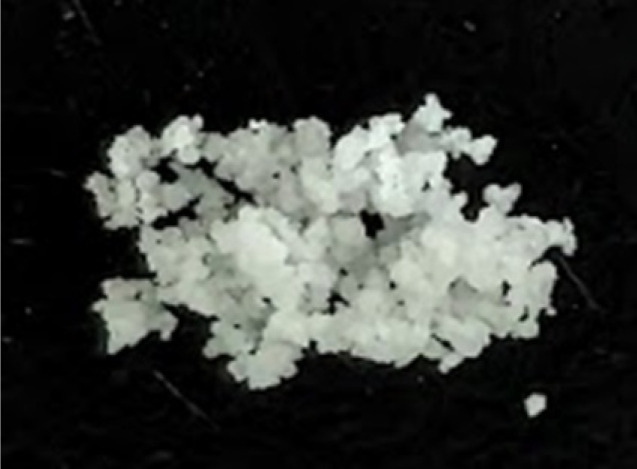
FD product obtained through freeze-drying, comprising solid-state cubic NH_3_(cr) as the main constituent.

Throughout the freeze-drying process, NH_3_(g)^28^ and BHO(g)^29^ were generated. These gaseous molecules were captured in an aqueous boric acid solution using a trap, as illustrated in Fig. 3. The trapped species could subsequently be utilised in the recycling process to produce FD products.

The phases present in the collected FD products were identified through a combination of techniques. X-ray diffraction (XRD) analysis was conducted using a Rigaku, Ultima IV instrument (Tokyo) while laser Raman spectroscopy was performed with a Nano Photon RAMAN Touch system (Osaka).

To determine the boron content within the FD products, inductively coupled plasma emission spectroscopy (ICP, Shimadzu, ICPS-8100, Kyoto) was employed.

The hydrogen and nitrogen contents were assessed utilising combustion thermal conductivity analysis (Elementar, VarioV MACRO, Yokohama).

For evaluating the upper storage temperature of NH_3_(cr), thermogravimetric analysis (TG, Rigaku, Thermoplus, TG 8110, Tokyo) was employed.

To analyse the contents of H_2_ and NH_3_ gases that evolved from the sample during thermal decomposition, gas and ion analyses were performed. Gas analysis was conducted using a J-Science lab GC7100 instrument (Kyoto), while ion analysis utilised a Thermo Fisher Scientific Integrion RFIC (Tokyo) system.

The penetration depth of X-rays, approximately 30 μm, allows for effective analysis of the structure of NH_3_(cr) embedded within the inner layer using XRD.^30^ However, the limitations of laser Raman spectroscopy lie in its restricted penetration depths, largely confined to the shallow layers near the surface due to the wavelength constraints of the laser. Consequently, the intensity of the spectrum originating from the inner layer tends to be weak. To supplement Raman spectroscopy, thermodynamic simulations were conducted to validate the formation of cubic ammonia.^31–36^

## Results and discussions

**Fig. 4:**
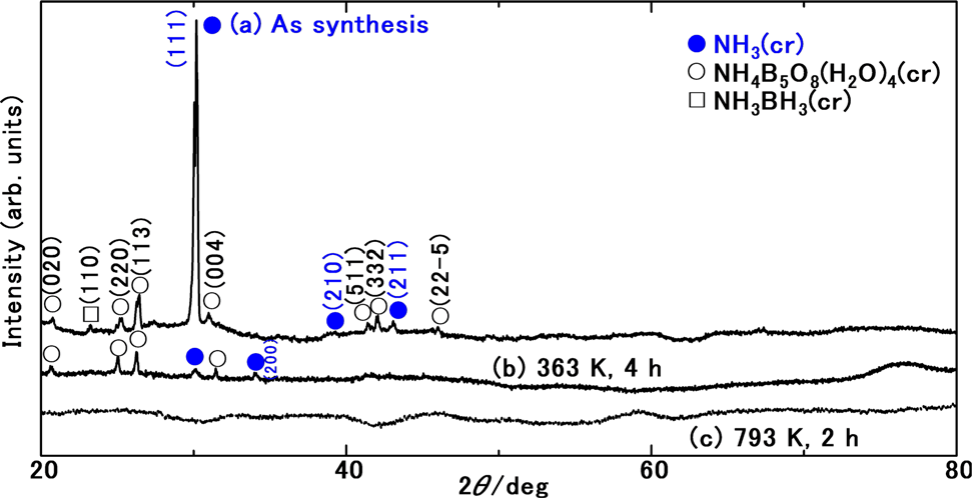
XRD patterns of reaction products comprising the solid-state cubic ammonia (NH_3_(cr)) as main product, ammonium pentaborate (NH_4_B_5_O_8_·4H_2_O(cr)) (open circle), and ammonia borane (NH_3_BH_3_(cr)) (open square) as the sub-products.

Fig. 3 presents the FD product, appearing as a white powder at 297 K under atmospheric pressure. Analysis of XRD patterns (Fig. 4) confirmed that the FD product consisted predominantly of solid-state cubic NH_3_(cr). In contrast to the commercial aqueous ammonia solution, which emits a pungent odour due to NH_3_(g) vaporisation, the odourless nature of the FD product, composed of NH_3_(cr), was noteworthy.

Fig. 4(a) depicts the XRD pattern of the FD product recorded at 297 K, revealing its main constituent as NH_3_(cr). The prominent peak observed at a 2*θ* value of 30.18° in this FD product aligned consistently with that of the solid-state cubic NH_3_(cr) detected at 171,^24,25^ 160,^26^ and 77 K (ref. 24 and 25) *via in situ* XRD conducted by Olovson and Templeton,^24,25^ as well as by Boese *et al.*^26^ Notably, the NH_3_(cr), which is naturally stable below 195.5 K under atmospheric pressure, was maintained at 297 K.

The lattice constant of the NH_3_(cr) in the FD product was determined through extrapolation of the diffraction data at a 2*θ* of 90.00°.^30^ Subsequently, utilising eqn (1), the lattice constants were computed for the (111), (210), and (211) planes at various diffraction angles denoted as 2*θ*°.1
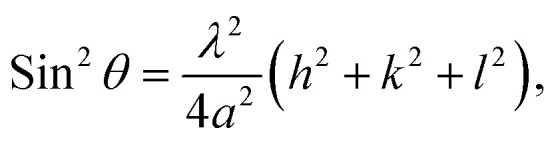


**Fig. 5:**
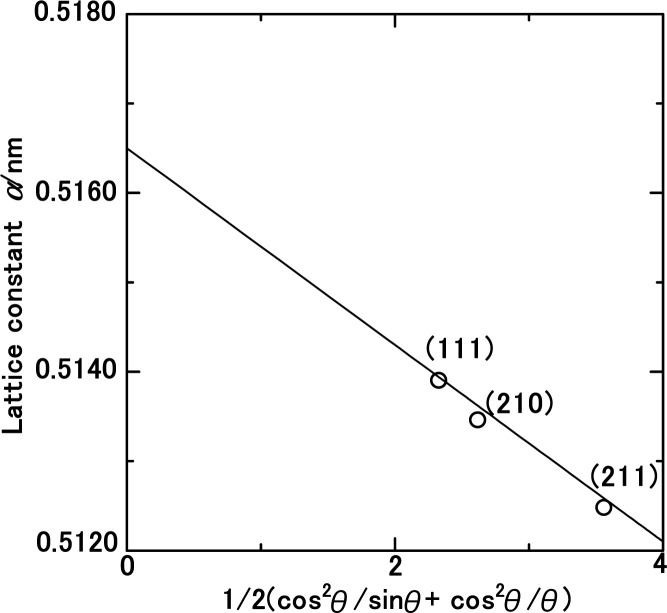
Lattice constant as function of the diffraction degree, *θ*, for the solid-state cubic ammonia (NH_3_(cr)) at 297 K.

In eqn (1) the Cu Kα-ray wavelength is 0.154056 nm, and *h*, *k*, and *l* represent the plane indices. As shown in Fig. 5, the lattice constants derived from the (111), (210), and (211) planes were plotted against 1/2(cos^2^ *θ*/sin *θ* + cos^2^ *θ*/*θ*), employing the least squares method, the lattice constant was extrapolated at a 2*θ* of 90.00° to ascertain its precise value.

Table 1 presents a comparative analysis of cubic lattice constants, denoted as ‘*a*’, for NH_3_(cr) as determined in this study alongside values obtained at various temperatures, namely 171,^24,25^ 160,^26^ and 77 K,^24,25^ through *in situ* XRD conducted by Olovson and Templeton,^24,25^ as well as by Boese *et al.*^26^ The lattice constant of 0.5165 nm identified in our investigation aligned closely with 0.5138,^24,25^ 0.51305,^26^ and 0.5084^24,25^ nm recorded at 171,^24,25^ 160,^26^ and 77 ^24,25^ K, respectively.

**Table 1:** Comparison of lattice constant, *a*, and the thermal expansion coefficient, *α*, for the synthesised solid-state cubic ammonia NH_3_(cr) at 297 K with values obtained at 77,^24,25^ 160,^26^ and 171 ^24,25^ K, as reported by Olovson and Templeton^24,25^ and Boese *et al.*,^26^ and *α* estimated from these data

Substance	*a*/nm	*α*	*T*/K	Remarks
NH_3_(cr)	0.5165	—	297	Present study
NH_3_(cr)	0.5138	—	171	24 and 25
NH_3_(cr)	0.51305		160	26
NH_3_(cr)	0.5084	—	77	24 and 25
NH_3_(cr)	—	1.26 × 10^−4^	171–297	Present study
NH_3_(cr)	—	3.34 × 10^−4^	77–160	24 and 26

To assess thermal expansion, the coefficient *α* was computed by differentiating the data between 297 K in our study and 171 K as per Olovson and Templeton.^24,25^ This was then compared with the findings reported by Olovson and Templeton^24,25^ and Boese *et al.*^26^ for the temperature range spanning from 160 (ref. 26) to 77 K,^24,25^ as detailed in Table 1. The resulting coefficients were determined to be 1.26 × 10^−4^ and 3.34 × 10^−4^, respectively.

These values are intricately linked to variations in the lattice vibration modes relative to temperature. Notably, larger coefficients are discernible within the lower temperature spectrum compared to those approximated around ambient conditions, aligning with both theoretical predictions^31^ and empirical evidence.^31–36^ The diffraction peaks from other planes of NH_3_(cr) were difficult to detect, being likely that X-ray intensity was decreased during penetrating GM. However, the determined lattice constant and thermal expansion constant is concluded to be reasonable.

Ammonium pentaborate tetrahydrate (NH_4_B_5_O_8_·4H_2_O(cr))^37^ and ammonia borane (NH_3_BH_3_(cr))^38–43^ are shown in Fig. 4(a). These include impurity phases stemming from the sub raw material B_2_O_3_(cr).

Shore and Böddeker^39^ demonstrated the preparation process of NH_3_BH_3_(cr), which involves several steps: (A) dispersing diborane (B_2_H_6_(g)) in tetrahydrofuran (THF) at 195 K to yield THF·BH_3_; (B) distilling liquid NH_3_(l) onto the THF solution followed by stirring; (C) distilling away NH_3_(g) and THF, subsequently extracting NH_3_BH_3_(cr) either from the remaining solid mixture of NH_3_BH_3_(cr) or diammoniate of diborane H_3_B(NH_3_)_2_^+^BH_4_^−^. Remarkably, the current process for producing the FD product closely mirrored the aforementioned synthesis of NH_3_BH_3_ at low temperatures by Shore and Böddeker.^39^

**Fig. 6:**
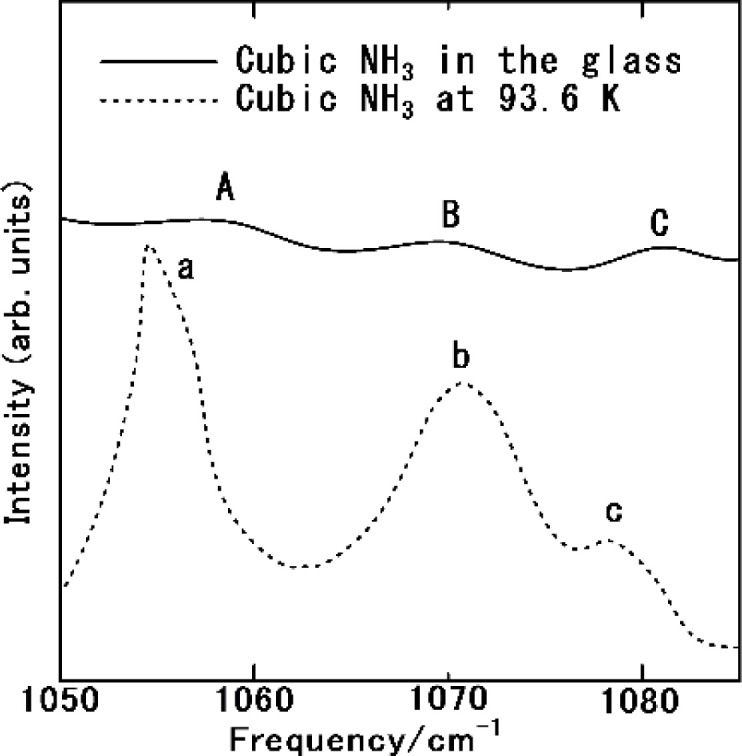
Raman spectrum of the cubic ammonia (NH_3_(cr)) confined in the glass matrix, compared with one measured by the *in situ* Raman spectroscopy by Nye and Medina at 93.6 K.^12^

Fig. 6 displays the Raman spectrum of NH_3_(cr) within the FD product, compared with one measured by the spectrum obtained *via in situ* Raman spectroscopy conducted by Nye and Medina at 93.6 K.^12^ The peaks A, B, and C observed in NH_3_(cr) within the FD product aligned consistently with the peaks a, b, and c detected in the low-temperature *in situ* measurements by Nye and Medina at 93.6 K.^12^ These peaks correspond to the Raman scattering spectra originating from the intermolecular vibrations of the ammonia molecules within the cubic unit cell, as illustrated in Fig. 1(a). Notably, the peak intensity of NH_3_(cr) was relatively low owing to confinement within the glass matrix, which limited the penetration of laser-induced Raman scattering.

Fig. 4(b) depicts the XRD pattern of the sample after maintaining the FD product following a duration of 4 h at 363 K. Notably, the disappearance of peaks corresponding to NH_3_(cr) signified sublimation into NH_3_(g), as described by reaction (I) in Table 2. Consequently, solely the peaks attributable to the remaining NH_4_B_5_O_8_·4H_2_O(cr) remained observable. This conversion of stored NH_3_(cr) into NH_3_(g) was notable for its lower energy consumption.

**Table 2:** Thermodynamic reactions for the related substances

NH_3_(cr) ⇌ NH_3_(g)	(I)
NH_4_B_5_O_8_·4H_2_O(cr) ⇌ NH_3_(g) + 4H_2_O(g) + 2B_2_O_3_(gl) + BHO(g) + 1/2O_2_(g)	(II)
B(OH)_3_(gl) ⇌ (1/2)B_2_O_3_(gl) + (3/2)H_2_O(g)	(III)
B_2_O_3_(gl) + B(OH)_3_(gl) ⇌ 3BHO(g) + 3/2O_2_(g)	(IV)

Fig. 4(c) illustrates the XRD pattern of the sample after subjecting the FD product at 793 K for 2 h. The absence of peaks associated with NH_4_B_5_O_8_·4H_2_O(cr) suggested its thermal decomposition according to reaction (II) in Table 2. Subsequently, a broad pattern emerged, revealing the presence of the remaining glass matrix (GM) containing the B_2_O_3_(gl) and B(OH)_3_(gl) components.

The thermal decomposition process of orthoboric acid B(OH)_3_(cr) unfolds in a series of steps.^44^ First, at 373 K, orthoboric acid B(OH)_3_(cr) undergoes decomposition to form metabolic acid HBO_2_(cr).^27^ Subsequently, at 413 K, metabolic acid HBO_2_(cr) further decomposes into tetra boric acid H_2_B_4_O_7_(cr).^44^ Finally, at 573 K, H_2_B_4_O_7_(cr) undergoes decomposition to yield boron trioxide B_2_O_3_(cr). Consequently, orthoboric acid B(OH)_3_(cr) transitions into B_2_O_3_(cr), as illustrated in reaction (III) in Table 2.

In a similar vein, a fraction of B(OH)_3_(gl) within the GM transforms into B_2_O_3_(gl), as depicted in reaction (IV) in Table 2, a transformation supported by the B, H, and N elemental analyses. Furthermore, interactions between B_2_O_3_(gl) and B(OH)_3_(gl) lead to the formation of gaseous BHO(g) and 3/2O_2_(g), a phenomenon also confirmed by elemental analyses.

Table 3 presents the elemental analysis results for B, determined using ICP, and for H and N, analysed *via* the combustion thermal conductivity. These assessments were conducted for both the FD product and the GM resulting from the thermal decomposition of the FD product at 793 K for 2 h. The values are expressed as mol%, with the O content derived by subtracting the sum of the B, H, and N contents from 100%. Notably, the FD product comprised 47.8 mol% H. In accordance with the sample preparation outlined earlier, 4.6 × 10^−3^ mol of both B and N were utilised, alongside 160 mg of B_2_O_3_(cr) powder placed in a test tube, followed by 29 mass% NH_3_(aq.). Consequently, 205 mg of the FD product was collected. Based on the chemical composition depicted in Table 2, the quantities of N and B in the 205 mg FD product were determined to be 1.6 × 10^−3^ and 3.0 × 10^−3^, respectively. It is noteworthy to consider that portions of N and B may have been lost as sublimed species, NH(g),^27^ and BHO(g),^29^ as illustrated in Fig. 3, which were captured as an aqueous ammonium–boric acid solution using a trap positioned between the FD vessel and vacuum pump.

**Table 3:** Chemical compositions of elements for the freeze-dried product (FD product) composed of solid-state cubic ammonia NH_3_(cr), glass matrix confining NH_3_(cr) (GM), calc. FD product, and calc. GM

	N/mol%	H/mol%	B/mol%	O/mol%	Remarks
FD	6.1	47.8	11.9	34.2	ICP & com.
GM	0.3	26.8	26.1	46.8	ICP & com.
Calc. FD	6.3	46.5	13.4	33.8	Estimation
Calc. GM	0	25.0	25.0	50.0	Estimation

Table 4 presents the molar percentage data detailing the phase constituents of both the FD and GM products, following a temperature hold at 793 K for 2 h. These estimates were derived from the elemental compositions outlined in Table 3. In the FD product, the molar percentages were determined as follows: 37 mol% NH_3_(cr), 5 mol% NH_4_B_5_O_8_·4H_2_O(cr), 10 mol% B_2_O_3_(gl), and 48 mol% B(OH)_3_(gl), labelled as calc. FD. Within this, the molar ratio of B_2_O_3_(gl) to B(OH)_3_(gl) was calculated as 0.17 : 0.83. Utilising mass percentage data, the composition revealed 11 mass% NH_3_(cr), 12 mass% NH_4_B_5_O_8_·4H_2_O(cr), 24 mass% B_2_O_3_(gl), and 53 mass% B(OH)_3_(gl), with a at B_2_O_3_(gl) to B(OH)_3_(gl) ratio of 0.31 : 0.69. Notably, B_2_O_3_(gl) acted as a glass-forming component, while B(OH)_3_(gl) dissolved within B_2_O_3_(gl), resulting in the formation of a glass structure.

**Table 4:** Estimated molar percentage data of constituents of freeze-dried product (calc. FD) comprising the solid-state cubic ammonia NH_3_(cr) and the components for GM after holding at 793 K for 2 h (calc. GM glass)

	Calc. FD/mol%	Calc. GM/mol%
NH_3_(cr) in FD	37	—
NH_4_B_5_O_8_·4H_2_O(cr) in FD	5	—
B_2_O_3_(gl) in FD	10	—
B(OH)_3_(gl) in FD	48	—
B_2_O_3_(gl) at 793 K for 2 h	—	50
B(OH)_3_(gl) at 793 K for 2 h	—	50

Elemental compositions were deduced from the molar percentage data, consistent with the calculated values for the FD product listed in Table 3. The composition of the calc. FD product aligned with the FD product, affirming the reasonableness of the molar percentage (Table 4). According to XRD (Fig. 4(a–c)) and TG (Fig. 8) analyses, NH_3_(cr), comprising 37 mol% of the FD product, sublimated into NH_3_(g) between 325 and 373 K, as expressed in reaction (I) in Table 2. NH_4_B_5_O_8_·4H_2_O(cr), constituting 5 mol% of the FD product, underwent thermal decomposition to NH_3_(g) above 373 K, as indicated in reaction (II) in Table 2. The resulting B_2_O_3_(gl) and B(OH)_3_(gl) were absorbed into the GM during the decomposition process. In the FD product, the molar ratio of B_2_O_3_(gl) to B(OH)_3_(gl) was 0.17 : 0.83. Therefore, the shift from a 0.83 ratio of B(OH)_3_(gl) to B_2_O_3_(gl) to a 0.43 ratio resulted in a phase composition similar to that of the GM consisting of 50 mol% B_2_O_3_(gl)–50 mol% B(OH)_3_(gl) after thermal decomposition at 793 K for 4 h. Nitrogen was detected in the elemental composition of GM after decomposition (Table 3). This residual N likely originated from the B–N bonding. However, the details of these coordination states remain unknown.

The quantities of the gaseous components were determined through the sublimation and thermal decomposition reactions outlined in reactions (I–IV) in Table 2. To calculate, we summed the masses of NH_3_(g) in reaction (I), H_2_O(g), BHO(g), and O_2_(g) in reaction (II), as well as H_2_O(g) in reaction (III), and BHO(g) and O_2_(g) in reaction (IV). The masses of the gaseous species in reactions (I–III) were assessed based on the molar percentages of the phase constituents. Upon adding the masses of BHO(g) and O_2_(g) in reaction (V), assuming an equivalent amount of the entire B_2_O_3_(gl) formed in the decomposition of the FD product was added to the sum of the masses of the gaseous species in reactions (I–III), we evaluated that 40.8 mass% of the initial FD product underwent gasification. Subsequently, the gasification mass loss during TG, after the completion of thermal decomposition, was determined to be 44.7%. Despite the complexity of the reactions involved, the calculated sum of mass loss during gasification aligned with the mass loss observed in TG. This consistency indicates an efficient conversion of NH_3_(cr) stored in the FD product (37 mol%) into NH_3_(g) within the temperature range of 325–373 K, with reduced energy consumption. H_2_(g) was separated using a hydrogen separation membrane. The solid-state cubic NH_3_(cr) synthesised in this study holds promise as an excellent hydrogen storage material. Future investigation will focus on determining the relative ratio of NH_3_(cr) to GM.

The phase stability of the NH_3_(cr) was investigated by computing its standard Gibbs energy of formation 
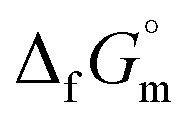
, with “standard” referring to thermodynamic values under 1 bar. Initially, the Gibbs energy of formation, Δ_f_*G*_m_, for NH_3_(g) at 195.5 K under 0.0609 bar—representing the temperature and pressure of the triple point—was calculated.^11–19^ Given that NH_3_(cr) is equilibrated with NH_3_(g) at the triple point, the Δ_f_*G*_m_ datum of NH_3_(cr) equals that of NH_3_(g). Subsequently, the 
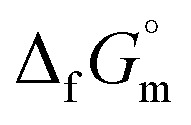
 datum at 298.15 K under 1 bar for cubic NH_3_(cr) was estimated.

The 
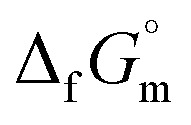
 datum for NH_3_(g) at 195.5 K under 1 bar was determined using eqn (2),2
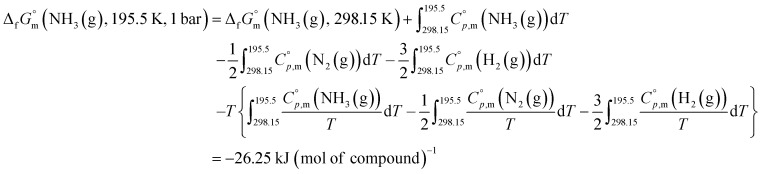
where 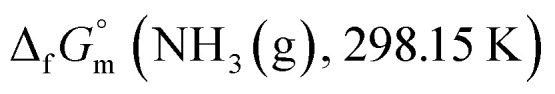 was taken from the thermodynamic state base (TDB) edited by Barlin (−16.41 kJ (mol of compound)^−1^),^27^ and the 
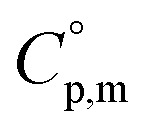
 data for NH_3_(g),^45^ N_2_(g),^29^ and H_2_(g)^29^ were adopted from the TDB reported by Wagman and Cox,^45^ and Chase *et al.*^29^

Similarly, the Δ_f_*G* datum of NH_3_(g) at 195.5 K under 0.0609 bar was calculated using eqn (3):3
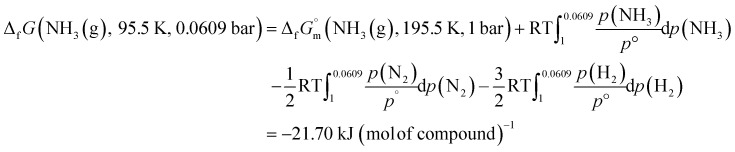


At the triple point, NH_3_(cr) is in equilibrium with NH_3_(g), establishing the equality of their Δ_f_*G*_m_ datum, as defined in eqn (4):

4 Δ_f_*G*(NH_3_(cr), 195.5 K, 0.0609 bar) = Δ_f_*G*(NH_3_(g), 195.5 K, 0.0609 bar) kJ (mol of compound)^−1^

The 
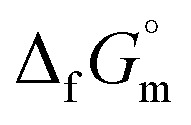
 datum for NH_3_(cr) at 195.5 K under 1 bar was determined using eqn (5), 5
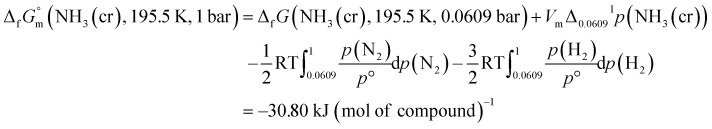
where the second term, *V*_m_Δ_0.0609_^1^*p*(NH_3_(cr)), is the increase of molar Gibbs energy of NH_3_(cr) with change in pressure. Because this term is negligible,^46^ it was not considered in the calculations.

Finally, the 
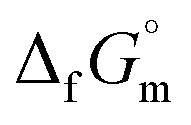
 datum of the cubic NH_3_(cr) at 298.15 K under 1 bar was calculated from eqn (6),6
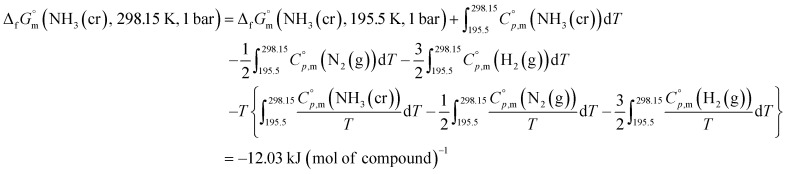
with estimated 
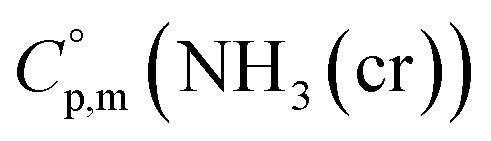
 data due to lack of direct measurement. The resulting value of 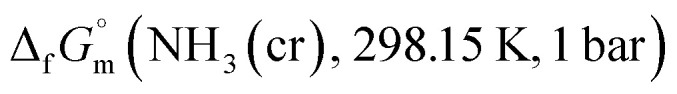 datum, determined as −12.03 kJ (mol of compound)^−1^, presents a novel finding, potentially impacting general science, owing to the quasi-equilibrium phase of cubic solid-state ammonia. The thermodynamic data were summarized in Table 5.

**Table 5:** Estimated thermodynamic data (TD) for NH_3_(cr) and NH_3_(g). Standard states are N_2_(g) and H_2_(g)

Phase	TD/kJ (mol of compd)^−1^		*T*/K	*p*/bar
Gas	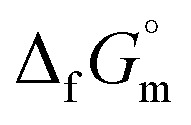 a	−16.41	298.15	1
Gas	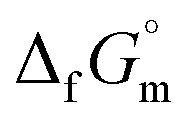	−26.25	195.5	1
Gas	Δ_f_*G*_m_	−21.70	195.5	0.0609
Solid	Δ_f_*G*_m_	−21.70	195.5	0.0609
Solid	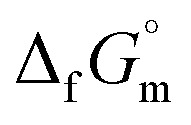	−30.80	195.5	1
Solid	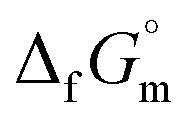	−12.03	298.15	1
—	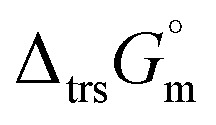 b	+4.38	298.15	1

a Ref. 27.

b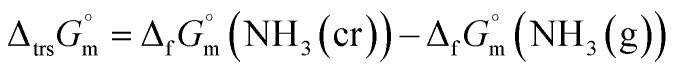.

Furthermore, the standard Gibbs energy of transition from the gas to cubic state 
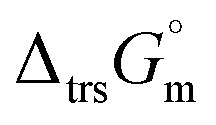
, at 298.15 K under 1 bar was calculated using eqn (7),7
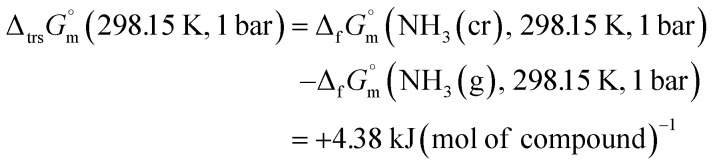


Hence, to stabilise NH_3_(cr) at 298.15 K, supplying the 
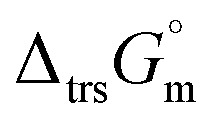
 datum is essential. A potential candidate is the phase boundary energy between NH_3_(cr) and GM, suggesting a strong N–B bond formation, which may stabilize the NH_3_(cr) structure at ambient pressure. Table S1 (see ESI)† shows the standard Gibbs energies of formation 
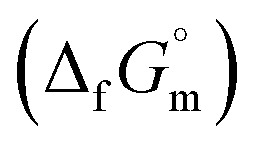
 for BN(cr),^27^ CrN(cr),^27^ and TiN(cr)^27^ related to the B–N bond along phase boundary between NH_3_(cr) and GM. Note that cubic BN(cr) having diamond like hardness is quasi equilibrium phase. Therefore, the 
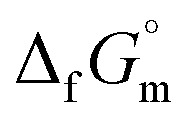
 datum^27^ of BN(cr) was determined for hexagonal structure as the equilibrium phase. The 
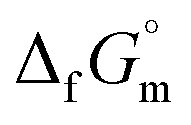
 data for CrN(cr)^27^ and TiN(cr)^27^ are deeply negative consistent with their high hardness due to strong covalency. As a result, they are used as coating materials to give wear resistance. The 
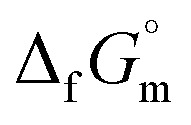
 datum of BN(cr)^27^ is intermediate value of ones of CrN(cr)^27^ and TiN(cr),^27^ meaning that the B–N covalent bond in its crystal is robust. From analogical reasoning, the B–N bonds along the phase boundary between NH_3_(cr) and GM appear to be robust. Another candidate is that the Gibbs energy of mixing, Δ_mix_*G*,^47,48^ indicating deeper equilibrium among NH_3_(cr), GM, and NH_4_B_5_O_8_·4H_2_O(cr) than with NH_3_(g). Additionally, the pressure from the embedded structure, particularly the compression pressure from the outer GM, appears to maintain the cubic structure of NH_3_(cr). Nonetheless, further investigation is warranted. The B–N bond states along the phase boundary between NH_3_(cr) and GM are likely to be further investigated by first principles calculation.^49^ The ternary quasi phase equilibria among NH_3_(cr)–GM–B_5_O_8_·4H_2_O(cr) is further investigated by the CALPHAD phase diagram calculation.^47,48^

The 
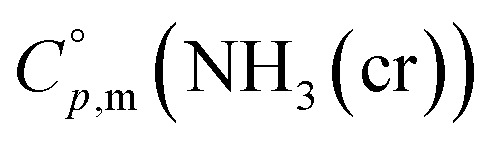
 data were derived by extrapolating from the data of solid-state hydrogen, H(cr),^50^ and nitrogen, N(cr),^50^ utilising the Neumann–Kopp law. At exceedingly low temperatures, the isochoric heat capacity, 
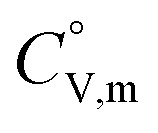
 can be closely approximated by eqn (8), as nearly all phonons predominantly occupy near-ground states, where ‘*n*’ represents the number of atoms in the formula unit.8
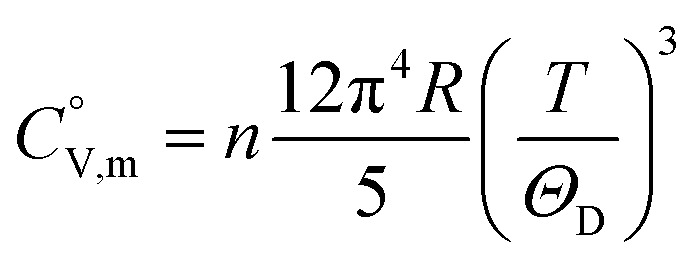


In eqn (8), *R* is the gas constant (=8.3145 (J K^−1^ mol^−1^)) and *Θ*_D_ is the Debye temperature. Consequently, at low temperatures, *Θ*_D_ should be independent of *T*.9
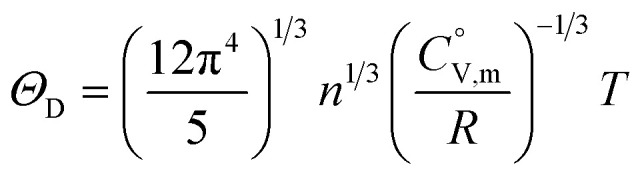


Because 
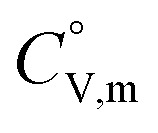
 and 
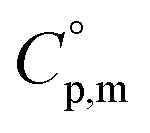
 exhibit striking similarity at extremely low temperatures,^33,34,36^*Θ*_D_ was derived from 
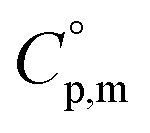
 values under 5 K and *T*, aligning with the relationship defined by eqn (9). *Θ*_D_ values for H(cr) and N(cr) were subsequently estimated as 141 K and 101 K, respectively.

**Fig. 7:**
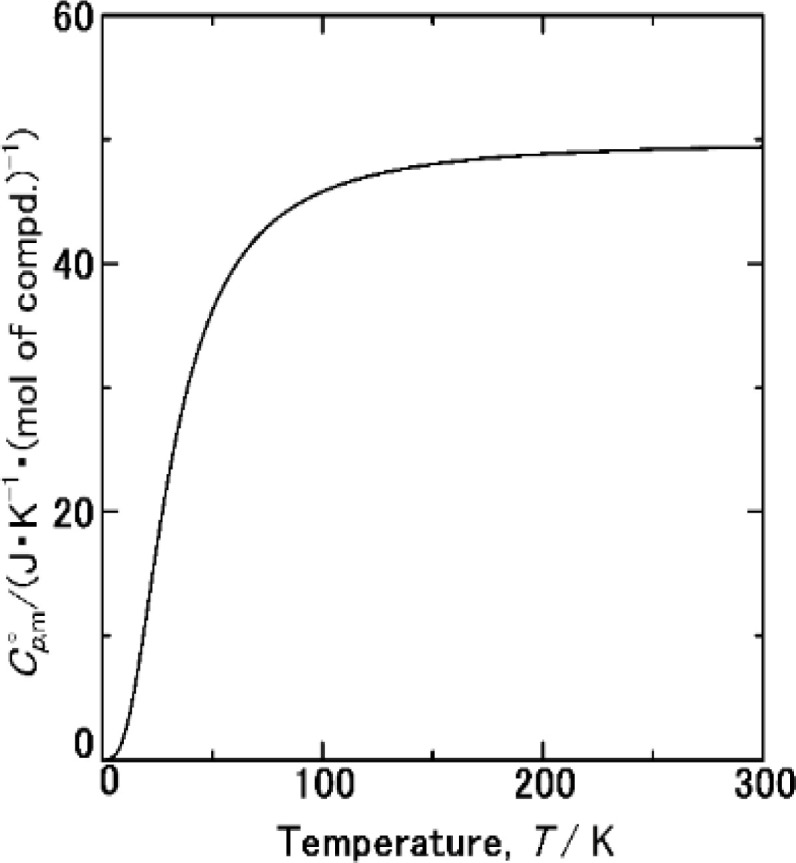
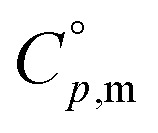
 data from thermodynamic simulation for solid state cubic NH_3_(cr) (Table S2†).

To extrapolate 
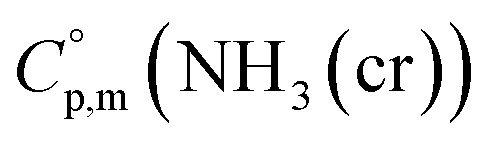
 across the 0–300 K range, a composite average of 
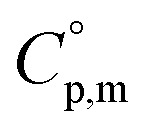
 data of H(cr) and N(cr) was employed, in accordance with the Neumann-Kopp law as shown in Fig. 7. Subsequent evaluation of the 
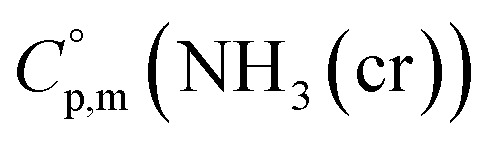
 data involved utilising recently developed formulas,^32–36^ detailed in the ESI (Table S2).† An optimised fitting function was then obtained and substituted into eqn (6) to ascertain 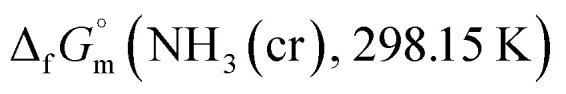.

**Fig. 8:**
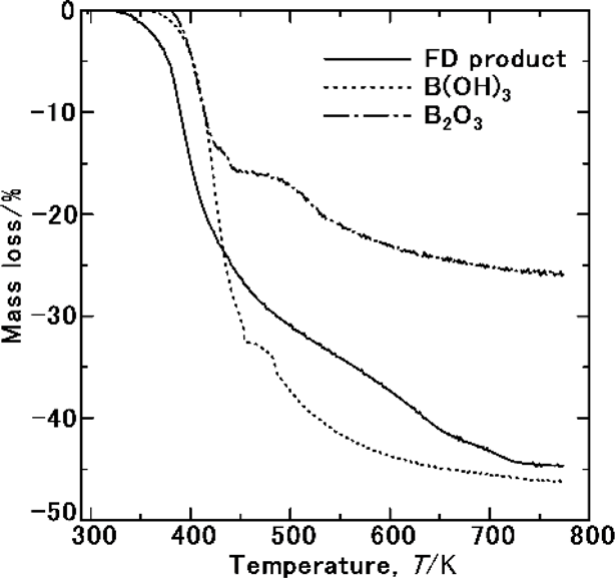
TG analyses of the samples prepared from freeze-drying (FD product), commercial B(OH)_3_(cr) and B_2_O_3_(cr), respectively. The initial masses of FD product, commercial B(OH)_3_(cr) and B_2_O_3_(cr) were 19.2, 20.6 and 13.7 mg, respectively.

Fig. 8 shows the TG results of the FD product used to investigate the sublimation of NH_3_(cr). No mass loss was observed below 325 K, indicating the possibility of the stable storage of the NH_3_(cr). At 325–750 K, mass loss occurred following reaction (I–IV) in Table 2 due to the sublimation of NH_3_(cr), the thermal decompositions of NH_4_B_5_O_8_·4H_2_O and B(OH)_3_ in GM, and forming BHO gas. The results of the TG data of B(OH)_3_ and B_2_O_3_ as GM forming elements were shown in Fig. 8. In TG of B(OH)_3_, mass loss occurred due to the thermal decomposition to form B_2_O_3_ and forming BHO gas. Since B_2_O_3_ is hygroscopic, its part is inevitably changed to form B(OH)_3_ absorbing atmospheric moisture. Therefore, the mass loss resulted from forming B(OH)_3_. However, the mole percents of the constituents in FD product was confirmed from B, H and N analyses by ICP spectroscopy and combustion thermal conductivity analysis. Considering hygroscopicity, utilization of B(OH)_3_ as starting substance is likely to be advantageous. This problem should be further investigated.

Finally, the investigation delved into the potential separation of hydrogen gas (H_2_(g)) from NH_3_(cr) confined in GM at 343 K. The process of sublimation posed challenges for the penetration of ammonia gas molecules through the GM layer at this temperature. Consequently, it is conjectured that NH_3_(cr) likely decomposes into hydrogen and nitrogen atoms, with subsequent penetration of H atoms through the GM layer, resulting in the formation of H_2_(g) on the surface. Conversely, N atoms are inferred to undergo a reaction with the B atoms in GM, leading to the formation of NH_4_B_5_O_8_·4H_2_O(cr).

To validate this hypothesis, a sample weighing 0.5 g of FD product was enclosed within a stainless-steel container and subjected to heating in a thermostatic bath at 343 K for 5 min, followed by holding for 6 h. Subsequently, the gas present in the stainless-steel container was collected in a gas bag post-cooling to room temperature. Analysis of the gas composition involved measuring the H_2_(g) content using a gas chromatograph. The residual gas was absorbed into a boric acid aqueous solution, and the content of NH_4_^+^(aq.) was determined using an ion chromatograph to quantify the presence of NH_3_(g).

Table 6 illustrates the quantities of H_2_(g) and NH_3_(g) following the FD product's exposure at 343 K for 6 h. Unfortunately, direct collection of H_2_(g) from the FD product was not feasible. However, NH_3_(g) was successfully detected, with a measured quantity of 8.9 mg per gram of FD product. This discovery underscores the efficient collection of NH_3_(g) at relatively low temperatures, signifying a promising avenue for energy conservation.

**Table 6:** Amount of H_2_(g) and NH_3_(g) after holding the FD product at 343 K for 6 h

Substance	Amount/mg (g-FD product)^−1^
H_2_(g)	None
NH_3_(g)	8.9

Nevertheless, a substantial amount of NH_3_(cr) is anticipated to remain sequestered within the GM, likely destined for conversion into NH_4_B_5_O_8_·4H_2_O(cr). This process indicates the potential for exploring alternative matrices with catalytic properties, such as zeolites^21^ and CaNH^22^ to facilitate the decomposition of internal NH_3_(cr) into H_2_(g) and N_2_(g). Thus, investigating outer matrices with catalytic capabilities represents an intriguing frontier for future research endeavours.

## Conclusion

Renewable energy sources such as solar photovoltaics and wind power are heavily reliant on weather and geography, necessitating complementary energy storage technologies. Ammonia serves as a crucial hydrogen storage substance, yet its gaseous form poses significant health risks, including potential fatality. Thus, safe ammonia storage systems must be developed. Our process design aimed to establish an embedded structure housing solid-state cubic ammonia (NH_3_(cr)) within a boric acid glass matrix. This innovative approach culminated in the successful synthesis of stable NH_3_(cr), preserved within the glass matrix under ambient temperature and pressure, facilitated by a freeze-drying process. Thermodynamic simulations validated the stabilisation of NH_3_(cr), with estimated standard Gibbs energy of formation affirming its stability in a quasi-equilibrium state. The pursuit of an outer matrix with catalytic capabilities, such as zeolites or CaNH, to facilitate the decomposition of inner NH_3_(cr) into H_2_(g) and N_2_(g), emerges as the next frontier in research.

## Author contributions

M. M. conceived the idea and wrote the paper; H. M. and H. K. synthesized the samples and analysed their constituents and compositions; and H. Y. rendered helpful discussions.

## Conflicts of interest

There are no conflicts to declare.

## Supplementary Material

RA-014-D4RA00229F-s001
