# Supplementary material for: Stabilisation of solid-state cubic ammonia confined in a glass substance at ambient temperature under atmospheric pressure
Source: RSC Adv. 2024 May 20;14(23):16128–37. doi: 10.1039/d4ra00229f (PMC11103458; doi:10.1039/d4ra00229f)
Supplement: RA-014-D4RA00229F-s001 [file RA-014-D4RA00229F-s001.pdf]

Electronic Supplementary Material (ESI) for RSC Advances.
This journal is © The Royal Society of Chemistry 2024

Supplementary material

Table S1 The standard Gibbs energies of formation ($\Delta_f G^\circ_m$) for BN(cr)[27], CrN(cr)[27], and TiN(cr)[27] related to the B−N bond along the phase boundary between NH_3(cr) and the B_2O_3 (gl)–$B(OH)_3$ (gl) glass matrix (GM).

Substance	$\Delta_f G^\circ_m$/(kJ (mol of compd.)$^{-1}$)
BN(cr)[27, *1]	-228.501
CrN(cr)[27]	-92.703
TiN(cr)[27]	-309.155

*1Hexagonal

The $C^\circ_{p,m}$ values for the solid state cubic NH_3(cr) were evaluated by the recently developed formulas:[32-34, 46,47] a semi-empirical power law function composed of the electronic and lattice vibration terms at $0 < T < 5\text{-}6$ K; an empirical power law temperature function at $5\text{-}6 < T < 40 - 50$ K; the Debye-Einstein (DE) at $40 - 50 < T < 300$ K, expressed as

$$C^\circ_{p,m} = \gamma T + \sum_{j=3,5,7,9} A_i T^i \qquad 0 < T < 5 - 6 \text{ K} \qquad \text{(S1)}$$

$$C^\circ_{p,m} = \sum_{j=0}^{6} B_j T^j \qquad 5 - 6 \text{ K} < T < 40 - 50 \text{ K} \qquad \text{(S2)}$$

$$C^\circ_{p,m} = 3\text{R}\left\{ mD\left(\frac{\Theta_D}{T}\right) + nE\left(\frac{\Theta_{E_1}}{T}\right) + lE\left(\frac{\Theta_{E_2}}{T}\right) \right\} \qquad 40 - 50 \text{ K} < T < 300 \text{ K} \qquad \text{(S3)}$$

where, for NH_3(cr), the fitting functions were divided in three temperature ranges: at 0.5 – 5.20 K; 5.20 – 41.36 K; 41.36 – 300 K, as shown in Tables S1-S3. At 0 – 5.20 K (eq.(S1)), γ is the coefficient of the electronic term contributing to $C^\circ_{p,m}$.[31-36] For insulating substances, it results from vacancies[32] A_i are the coefficients for the lattice vibration term contributing to $C^\circ_{p,m}$.[29-34] At 5.20 – 41.36 K (eq.(S2)), B_i are the adjustable coefficients for providing sufficient overlap to the measured $C^\circ_{p,m}$ data and for connecting smoothly near the inflection points around 5.02 K and

41.36 K. At 41.36 – 300 K (eq.(S3)), $\left(\frac{\Theta_D}{T}\right)$, $\left(\frac{\Theta_{E_1}}{T}\right)$ and $\left(\frac{\Theta_{E_2}}{T}\right)$ are Debye and Einstein functions; Θ_D, Θ_{E_1} and Θ_{E_2} are the Debye and Einstein temperatures used for adjustable parameter; *m*, *n* and *l* are the adjustable parameters and the sum of *m*, *n* and *l* should be approximately closed to the number of atoms in the formula unit. [33-36]. Tables S1-S3 shows the adjustable parameters. Six digits numbers for Θ_D, Θ_{E_1}, Θ_{E_2}, *m*, *n* and *l* are necessary to reproduce the experimental $C_{p,\,m}^{\circ}$ data as five digits numbers.

The $C_{p,\,m}^{\circ}$ data at 298.15 K of metal elements are about 25 (J K^{-1} (mol of atoms)$^{-1}$) following the Dulong-Petit law, and the Debye and Einstein functions are the theories[(29)] to satisfy the Dulong-Petit law. However, the compounds composed of non-metallic element do not follow the Dulong-Petit law, and their $C_{p,\,m}^{\circ}$ data are empirically half values, e.g. the $C_{p,\,m}^{\circ}$ data at 298.15 K of B_2O_3(cr) and $B(OH)_3$ are 62.761 (J K^{-1} (mol of compd.)$^{-1}$), i.e. , 12.552 (J K^{-1} (mol of atoms)^{1}), and 86.060 (J K^{-1} (mol of compd.)$^{-1}$), i.e. , 12.294 (J K^{-1} (mol of atoms)$^{-1}$, respectively. Therefore, the $C_{p,\,m}^{\circ}$ data for NH_3(cr) were adopted as the half values calculated from the parameters summarized in Table S2.

Table S2 Parameters for the fitting functions used to fits the $C_{p,m}^{\circ}$ data for the solid state cubic NH_3(cr) from Eqs.S1-S3. The notation E $\pm$ *kl* indicates the power of 10.

Temp. range: 0.5 – 5.20 K	Coefficient
γ(J K^{-2} (mol of compd.)$^{-1}$	8.07997E-08
A_3(J K^{-4} (mol of compd.)$^{-1}$	1.98334E-03
A_5(J K^{-6} (mol of compd.)$^{-1}$	2.67852E-08
A_7(J K^{-8} (mol of compd.)$^{-1}$	-3.48652E-09
A_9(J K^{-10} (mol of compd.)$^{-1}$	1.93042E-10
A_{11}(J K^{-12} (mol of compd.)$^{-1}$	-3.81599E-12
**Temp. range: 5.20 – 44.29 K**	**Coefficient**
B_0/(J K^{-1} (mol of comd.)$^{-1}$)	-2.49757E-01
B_1/(J K^{-2} (mol of comd.)$^{-1}$)	2.18229E-01
B_2/(J K^{-3} (mol of comd.)$^{-1}$)	-6.30746E-02

B_3/(J K^{-4} (mol of comd.)$^{-1}$)	9.77004E-03
B_4/(J K^{-5} (mol of comd.)$^{-1}$)	-4.12224E-04
B_5/(J K^{-6} (mol of comd.)$^{-1}$)	7.37473E-06
B_6/(J K^{-7} (mol of comd.)$^{-1}$)	-4.92858E-08
Temp. range: 44.29 – 300 K	Coefficient
Θ_D/K	1.10961.E+02
m	6.20324E-01
Θ_{E1}/K	5.32855E+03
n	1.37968E+00
Θ_{E2}/K	1.31707E+02
l	2.00000E+00
